# Complete genome sequence, metabolic model construction, and *huangjiu* application of *Saccharopolyspora rosea* A22, a thermophilic, high amylase and glucoamylase actinomycetes

**DOI:** 10.3389/fmicb.2022.995978

**Published:** 2022-09-28

**Authors:** Donglin Ma, Shuangping Liu, Xiao Han, Mujia Nan, Yuezheng Xu, Bin Qian, Lan Wang, Jian Mao

**Affiliations:** ^1^State Key Laboratory of Food Science and Technology, National Engineering Research Center of Cereal Fermentation and Food Biomanufacturing, School of Food Science and Technology, Jiangnan University, Wuxi, China; ^2^Shaoxing Key Laboratory of Traditional Fermentation Food and Human Health, Jiangnan University (Shaoxing) Industrial Technology Research Institute, Shaoxing, China; ^3^National Engineering Research Center of Huangjiu, Zhejiang Guyuelongshan Shaoxing Wine Co., Ltd., Shaoxing Huangjiu Industry Innovation Service Complex, Shaoxing, China; ^4^Basic Department, University of Tibetan Medicine, Lhasa, China

**Keywords:** *Saccharopolyspora rosea*, whole-genome sequence, genome-scale metabolic model, stress resistance, *huangjiu* fermentation

## Abstract

*Saccharopolyspora* is an important microorganism in the fermentation process of wheat *qu* and *huangjiu*, yet the mechanisms by which it performs specific functions in *huangjiu* remain unclear. A strain with high amylase and glucoamylase activities was isolated from wheat *qu* and identified as *Saccharopolyspora rosea* (*S. rosea*) A22. We initially reported the whole genome sequence of *S. rosea* A22, which comprised a circular chromosome 6,562,638 bp in size with a GC content of 71.71%, and 6,118 protein-coding genes. A functional genomic analysis highlighted regulatory genes involved in adaptive mechanisms to harsh conditions, and *in vitro* experiments revealed that the growth of *S. rosea* A22 could be regulated in response to the stress condition. Based on whole-genome sequencing, the first genome-scale metabolic model of *S. rosea* A22 named *i*SR1310 was constructed to predict the growth ability on different media with 91% accuracy. Finally, *S. rosea* A22 was applied to *huangjiu* fermentation by inoculating raw wheat *qu*, and the results showed that the total higher alcohol content was reduced by 12.64% compared with the control group. This study has elucidated the tolerance mechanisms and enzyme-producing properties of *S. rosea* A22 at the genetic level, providing new insights into its application to *huangjiu*.

## Introduction

The composition of the microbial community during the fermentation of *huangjiu*, mainly derived from wheat *qu*, has a crucial relationship with the flavor of *huangjiu* (Huang et al., 2018, 2019). Wheat *qu*, which contains amylase, glucoamylase, protease, and cellulase, is an important starter used in *huangjiu* brewing (Xu et al., 2016; Chen et al., 2019). Genome sequencing showed that the abundance of *Saccharopolyspora* in *huangjiu* fermentation and Shaoxing wheat *qu* ranged from 18 to 21% (Wang et al., 2014; Zhang et al., 2022), and also indicated that *Saccharopolyspora* in *huangjiu* fermentation may originate from wheat *qu* (Liu et al., 2019). *Saccharopolyspora* was involved in the synthesis of flavor compounds such as esters, acids, and phenols in *huangjiu* fermentation based on metagenomic function prediction (Liu et al., 2019). Therefore, there is a need to understand the genomic information of *Saccharopolyspora* and how it relates to the fermentation of *huangjiu*.

*Saccharopolyspora* was first described by Lacey and Goodfellow (1975), and was a safe class of bioresource bacteria (Sayed et al., 2020). Up to now (June 30, 2022), the names of 36 species derived from the genus *Saccharopolyspora* were effectively published.^1^ The genome of 21 of these species has been uploaded to the NCBI database,^2^ but the genome of *Saccharopolyspora rosea* is missing. With the development of high-throughput sequencing technology, emerging sequencing platforms including Illumina, Pacbio, and Nanopore (Loman et al., 2015) are widely used in genome sequencing. The first genome of *Saccharopolyspora erythraea* NRRL23338 was completed in 2007, which led to the first in-depth analysis of *Saccharopolyspora* at the genetic level (Oliynyk et al., 2007). Further, comparative genomic analysis was employed to enhance the amylolytic enzymes by overexpression of genes *glnR* and *phoP* encoding α-amylase, glucoamylase, and α-glucosidase, resulting in enhanced carbohydrate utilization (Xu et al., 2016). The key genes for low urea production in *Saccharomyces cerevisiae* JH301 were analyzed and verified by whole genome sequencing, which provided new insight for controlling ethyl carbamate in *huangjiu* (Liang et al., 2022). Genome-scale metabolic models (GSMM) reconstructed from genome-wide data have novel applications in microbiology. GSMM predicts the cellular metabolism of organisms through metabolites and metabolic fluxes and is also used to analyze and optimize microbial growth and biological product production (Xu et al., 2018). Remarkably, KBase, a web-based resource^3^ can be used directly to construct GSMMs between eukaryotes and prokaryotes, and to explore microbial phenotypic characteristics (Arkin et al., 2018).

In this study, a strain with high amylose was isolated and identified as *Saccharopolyspora rosea* A22 (*S. rosea* A22) from wheat *qu*. We sequenced the complete genome of *S. rosea* A22, which provided comprehensive insight into the enzyme production characteristics, metabolic diversity, and functional features. A functional genomic analysis highlighted tolerance-related genes (temperature, osmotic stress, salt, and pH), while additional experiments clarified the tolerance mechanism of strain A22 in wheat *qu* and *huangjiu* fermentation. Meanwhile, the GSMM of *S. rosea* was constructed based on whole-genome sequencing to predict its ability to utilize different nitrogen sources. Finally, the potential of this strain in *huangjiu* fermentation was also validated. Together, these findings provide insights into the potential of *S. rosea* A22 for its application in *huangjiu*, and the flow chart of the entire work was shown in Supplementary Figure S1.

## Materials and methods

### Isolation and screening of the enzyme-producing strain

Wheat *qu* and fermenting *huangjiu* mash were collected from a *huangjiu* factory in Shaoxing (30°08’ N, 120°40′9 E), Zhejiang Province, China in July 2020. All samples were serially diluted with sterile water and spread on actinomycetes medium (KNO_3_ 1.0 g/l, KH_2_PO_4_ 0.5 g/l, MgSO_4_ 0.5 g/l, FeSO_4_ 0.01 g/l, NaCl 0.5 g/l, starch 20.0 g/l, Agar 15.0 g/l) for incubation at 37°C for 5–7 days. The isolated strains were spotted in triplicate on actinomycetes medium for incubation at 37°C for 5–7 days, and then the plates were flooded with 1 ml of iodine solution for amylase activity assays. The enzyme activity index (EI) was calculated as the ratio of the mean diameter of substrate degradation to the mean diameter of the colony (Zhang et al., 2021).

### 16𝑆 rRNA gene sequencing and phylogenetic analysis of strain A22

Genomic DNA was extracted from cultures incubated at 37°C for 72 h using a bacterial DNA extraction kit (Sangon Biotech, Shanghai, China). The 16S rRNA gene was amplified by PCR using two universal primers, including primers 27F (5′- AGAGTTTGATCCTGGCTCAG-3′) and 1492R (5’-TACGGTTACCTTGTTACGACTT-3′) (Liang et al., 2014). The 25 μL mixtures were composed of 1 μL template DNA, 12.5 μL of 2 × Taq PrimerSTAR HS (R040A), 1 μL of each primer (10μM), and 9.5 μL of double-distilled H_2_O. The PCR program was 98°C for 1 min, followed by 30 cycles of 98°C for 30 s, 60°C for 30 s, and 72°C for 1.5 min, with a final 5 min extension at 72°C and completion at 4°C. The PCR product was analyzed by electrophoresis in 2% agarose gel and then sent to Sangon Company (Shanghai, China) for sanger sequencing using an ABI sequencer (3730xl DNA Analyzer). The phylogenetic tree was constructed by applying the neighbor-joining method using the MEGA 11 software package (Temple University, PA, United States) based on Maximum Composite Likelihood with 1,000 replicates of bootstrap values.

### Morphological, physiological, and biochemical identification of *Saccharopolyspora rosea* A22

The morphological characteristics of *S. rosea* A22 were studied using macroscopic and microscopic features. The morphological properties of *S. rosea* A22, including shape, spore chains, and colony characteristics (color, shape, surface, elevation, and edge) were evaluated (Kim and Goodfellow, 2015). The physiological and biochemical characterization of *S. rosea* A22 was performed by using a bacterial biochemical identification tube (hopebio, Qingdao, China).

### Stress resistance analysis

The tolerance of *S. rosea* A22 was investigated, including ethanol, reducing sugar, and lactic acid, and the ability to grow under different temperatures. Ethanol tolerance was investigated by weighing the weight of mycelium in actinomycetes medium supplemented with 2% (v/v), 4% (v/v), 6% (v/v), 8% (v/v), and 10% (v/v) ethanol. Tolerance of lactic acid and reducing sugars were evaluated by varying concentrations of lactic acid (2, 4, 6, and 8 g/l) and reducing sugars (30, 50, 70, and 90 g/l). Temperature tolerance was investigated by weighing the weight of the mycelium of *S. rosea* A22 cultured at 37°C, 45°C, and 50°C, respectively. All fermentation experiments were performed in sealed 250 ml blue cap bottles with triplicates. The dry weight of the medium without *S. rosea* A22 was also used as a control to determine the actual dry weight of the mycelium under different harsh conditions.

### Genome sequencing, assembly, and annotation

Genomic DNA was extracted from cultures incubated at 37°C for 48–72 h using a bacterial DNA extraction kit (Sangon Biotech, Shanghai, China). DNA integrity was evaluated by agarose gel electrophoresis, and a Qubit® 2.0 Fluorometer (Thermo Fisher Scientific) was employed to measure the DNA quantity and quality. The whole genome library of *S. rosea* A22 was produced using the Nanopore PromethION platform (Oxford Nanopore Technologies Ltd., Oxford, United Kingdom) and Illumina Navaseq PE150 (Illumina, SanDiego, CA, United States) at the Novogene Company (Tianjin, China). Genome assembly was employed using Unicycler software (Version 0.4.8) (Wick et al., 2017). The GeneMarkS (Version 4.17) was used to predict the related coding gene. The databases gene ontology (GO), Kyoto encyclopedia of genes and genomes (KEGG), cluster of orthologous groups of proteins (COG), and carbohydrate-active enZYmes (CAZy) were used to predict gene functions.

### Genome-scale metabolic modeling of *Saccharopolyspora rosea* A22

#### Model reconstruction

The whole genome of *S. rosea* A22 was uploaded to KBase, a web-based resource (see text footnote 3) and was annotated using the function “Annotate Genome/Assembly with RASTtk.” A draft model was constructed based on the “Build Metabolic Model” function in KBase using annotated genome-wide information. The KBase tool adapts the original Escher web application to visualize metabolic models (Rowe et al., 2018).

#### Model curation

Metabolic models were constructed using the automated pipeline KBase and some metabolic reactions and compounds were missed due to genome annotation errors. *S. rosea* can utilize a variety of carbon and nitrogen sources in Bergey’s Manual of Systematics of Archaea and Bacteria (Kim and Goodfellow, 2015). The draft model of *S. rosea* was gapped using different synthetic media to automate the addition of compounds and reactions. Finally, the “check model mass balance” function in KBase was used to check the mass balance on both sides of the refined model.

#### Model evaluation and validation

In this study, model predictions and ‘wet experiments’ were used to verify the reliability of the model. Based on the physicochemical properties of *S. rosea*, it was found that the main carbon sources used were galactose, arabinose, mannose, mannitol, arabinitol, xylose, glucose, starch, sorbitol, and the main nitrogen sources used were tyrosine, urea, phenylalanine, histidine, hypoxanthine and casein. Qualitative experiments were performed by using these substrates as the only carbon or nitrogen sources to verify the reliability of the model.

#### Application of *Saccharopolyspora rosea* A22 to *huangjiu* fermentation

The raw wheat *qu* inoculated with *S. rosea* A22 was prepared by the previous methods (Ma et al., 2022). Briefly, the activated *S. rosea* A22 seed solution was mixed with water to adjust the bacterial concentration to 10^6^ CFU/ml, then the bacterial suspension was added to the crushed wheat at 25–26% (v/m), stirred well and transferred to the fermentation room for 120 h at 45°C. The saccharification solution was prepared by referring to the previous method (Wei et al., 2017; Liu et al., 2021a) and was employed to expand the seed solution of *Saccharomyces cerevisiae* HJ at 28°C, 150 rpm/min for 36 h. *Huangjiu* fermentation process was modified referring to the previous method by replacing all the wheat *qu* with inoculated raw wheat *qu* except for the control group (Wei et al., 2017). Total acid and amino nitrogen were determined by using titration (Gump et al., 2002). Reducing sugar content was determined by using the dinitro salicylic acid (DNS) method (Zohri et al., 2018). The ethanol concentration and higher alcohol content were determined as previously described (Vilanova et al., 2012; Wei et al., 2017). The HPLC conditions used to analyze free amino acids were the previous method (Wang et al., 2014). Higher alcohols and volatile flavor compounds were determined by the previous method (Liu et al., 2019).

#### Data analysis

One-way analysis of variance (ANOVA) was carried out to evaluate all data for significant differences (*p* < 0.05) using SPSS 19.0 software, and GraphPad prism for image processing.

## Results and discussion

### Isolation and identification of amylase-producing strains

**Figure 1:**
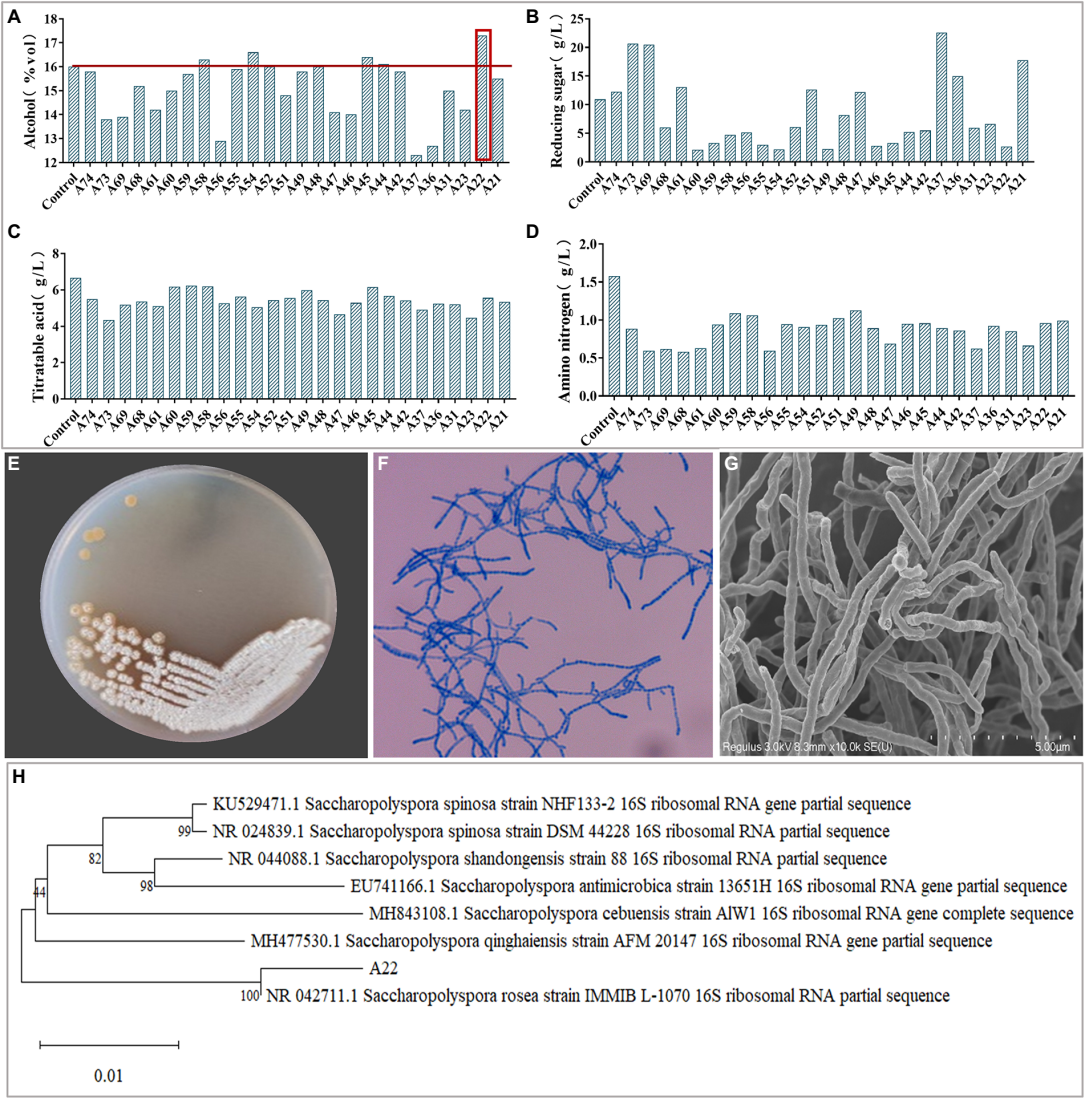
**(A–D)** The ethanol, reducing sugar, titrating acid and amino nitrogen content of *Huangjiu* fermented by raw wheat *Qu* inoculated with different strains, respectively. Colony morphology in plates **(E)**, microscopy (400 ×) **(F),** scanning electron micrographs (10.0 k ×) **(G)**, and phylogenetic tree **(H)** of *Saccharopolyspora rosea* A22.

Plate color reaction indicated that 75 strains with amylase production capacity were isolated (Supplementary Table S1), and we screened 26 strains with EI > 20 for inoculated raw wheat *qu* and *huangjiu* fermentation experiments. Results showed that there were differences in the physicochemical parameters including ethanol content, titratable acids, reducing sugars, amino nitrogen (Figures 1A–D), and the *huangjiu* fermented with raw wheat *qu* inoculated with strain A22 had a higher alcoholic content (17.30%vol) than the other experimental groups (Figure 1A). At the same time, we determined the glucoamylase and amylase of six kinds of inoculated raw wheat *qu* with an alcohol content higher than 16%vol (Supplementary Figure S2). The results showed that the glucoamylase (1,314 U/g) and amylase (1.41 U/g) of raw wheat *qu* inoculated with strain A22 were significantly (*p* < 0.05) higher than those of other experimental groups, indicating that the strain A22 produced high glucoamylase and amylase. Further, we performed colony morphology, physical and chemical characteristics, and molecular identification on strain A22. The strain A22 can produce pink-colored diffusible pigments, form branched brownish-yellow substrate hyphae, and yellowish to white aerial hyphae that differentiate into long straight chains of smooth-surfaced spores (Figures 1E–G). The strain A22 can grow at 28–50°C and pH 4.0–10 (Table 1). The carbon sources that the strain A22 can utilize were arabinose, galactose, mannitol, xylose, sucrose, starch, and sorbitol, but not raffinose, rhamnose, fructose, lactose, and maltose. In addition, L-phenylalanine, L-histidine, urea, hypoxanthine, nitrate, hesperidin, and tyrosine can be used as the sole source of nitrogen by strain A22, but xanthine and L-valine cannot be utilized. These results were consistent with the description of *Saccharopolyspora rosea* in Bergey’s Manual of Systematics of Archaea and Bacteria.

**Table 1:** Differential physiological properties of *Saccharopolyspora rosea* A22.

Carbon source	Growth	Nitrogen source	Growth	Salt tolerance	Growth	Temperature (°C)	Growth
Arabinose	+	L-valine	−	0.1%	+++	4	−
Raffinose	−	L-phenylalanine	+	1%	++	28	+
Rhamnose	−	L-histidine	++	4%	++	37	+++
Lactose	−	Urea	+	7%	+	45	++
Galactose	+	Hypoxanthine	+++	10%	−	50	+
Mannitol	+	Xanthine	−				
Xylose	+	Nitrate	++			pH	
Sucrose	++	Esculin	+			4	+
Fructose	−	Tyrosine	+			7	+++
Starch	+++					10	+
Sorbitol	++						
Maltose	−						

“+,” growth; “++,” good growth; “+++,” great growth; “−,” no growth.

The phylogenetic tree of strain A22 was constructed based on 16S rRNA sequence alignment, and it was found that strain A22 was 100% similar to *Saccharopolyspora rosea* IMMIB L-1070 (KY108649.1) (Figure 1H). This phylogenic relationship showed that strain A22 belongs to the *Saccharopolyspora* genus, and we named strain A22 as *Saccharopolyspora rosea* A22.

### General characteristics of the complete genome of strain *Saccharopolyspora rosea* A22

The complete genome of *S. rosea* A22 was sequenced and shown in Table 2. The whole-genome sequence of *S. rosea* A22 comprised a circular chromosome 6,562,638 bp in size and a GC content of 71.71%, and 6,118 protein-coding genes (CDS) (Table 2). The total length of the CDS was 5,733,141 bp, accounting for 87.35% of the total genome length. Furthermore, 12 rRNA and 52 tRNAs were identified by rRNAmmer (Version 1.2) (Nawrocki et al., 2015) and tRNAscan-SE (Version 1.3.1) (Besemer et al., 2001), respectively.

**Table 2:** General feature of the genome of *S. rosea* A22.

Item	Description
Genome size (bp)	6,562,638
Chromosome	1
Topology	circular
N50 length (bp)	6,562,638
L50	1
Protein-coding genes (CDS)	6,118
GC content (%)	71.71
Gene length (bp)	5,733,141
Gene average length (bp)	937
Gene internal length (bp)	829,497
Number of tRNA genes	52
Number of rRNA genes	12

### Functional analyses of the complete genome of strain *Saccharopolyspora rosea* A22

**Figure 2:**
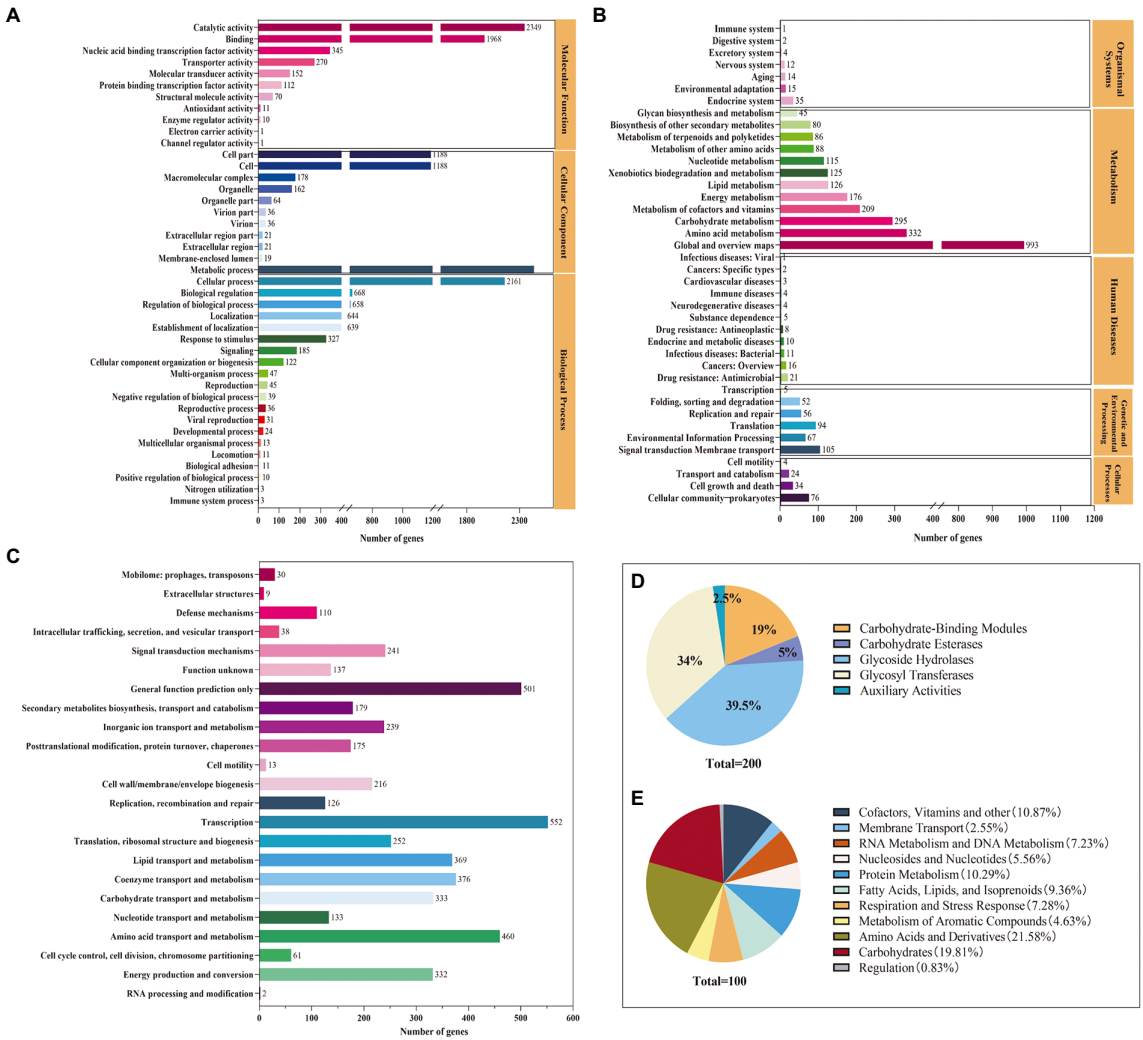
Genomic functional annotation of *S. rosea* A22 **(A)** GO annotation, **(B)** KEGG annotation, **(C)** COG annotation, **(D)** CAZy functional categories, and **(E)** subsystems distribution of *S. rosea* A22 based on RAST server annotation).

The functional annotation indicated that 4,057, 4,886, 3,355, and 200 genes were annotated in the GO, COG, KEGG, and CAZys databases, respectively (Figure 2). A total of 21 functional categories, including metabolic process, cellular process, and localization, were annotated in the biological process, with metabolic process and cellular process accounting for 30 and 27% of the genes annotated in the biological process (Figure 2A). Eleven functions were annotated in the molecular function classification, with the most annotated functions catalytic activity and binding, accounting for 44 and 37% of molecular functions, respectively. KEGG can intuitively analyze the relationship between genes and compounds in metabolic pathways. The Level 1 metabolic pathways were annotated in the categories of organismal systems (2.47%), metabolism (78.24%), human diseases (2.53%), genetic information processing (6.17%), environmental information processing (5.13%), and cellular processes (4.11%) (Figure 2B). Among the Level 2 metabolic pathways, global and overview maps, amino acid metabolism, carbohydrate metabolism, cofactor, and vitamin metabolism were the most annotated genes with 993, 332, 295, and 209, respectively. The coding proteins identified by *S. rosea* A22 accounted for 23 functional classifications in the COG database (Figure 2C). Among the groups, general function prediction only (10.25%), amino acid transport and metabolism (9.41%), coenzyme transport and metabolism (7.69%), carbohydrate transport and metabolism (6.82%), energy production and conversion (6.79%) were the main categories annotated. A total of 200 CAZymes were annotated in *S. rosea* A22, and these CAZymes belonged to 6 protein families, of which GH was the most abundant, accounting for 39.50% of the total genes (Figure 2D). Glycoside hydrolases (GH) are a class of enzymes that can catalyze the hydrolysis of glycosidic bonds in carbohydrates (De Benedetti et al., 2020). In the GH family, small gene families GH23, GH25, and GH15 annotated 16, 4, and 4 genes, respectively. The *S. rosea* A22 genome also includes 68 genes encoding glycosyl transferases (GT), 38 genes encoding carbohydrate-binding modules (CBM), 10 genes encoding carbohydrate esterases (CE), and 5 genes encoding auxiliary activities (AA). GT is a class of enzymes that can catalyze most glycosyl transfer reactions (Vasudevan and Lee, 2020), and A22 has annotated 68 related genes, accounting for 34% of the total. In addition, the subsystem distribution based on the RAST annotation^4^ indicates that cofactors, vitamins, and others (10.86%), amino acid metabolism (21.58%), protein metabolism (10.29%), carbohydrate metabolism (19.81%), and fatty acids, lipids, and isoprenoids (9.36%) are the major metabolisms (Figure 2E).

### Potential of *Saccharopolyspora rosea* A22 for hydrolysis of starch and proteins in *huangjiu* fermentation

Alpha-amylase (EC.3.2.1.1) can break the α-1,4-glycosidic bond of starch, glycogen, or polysaccharides to produce products such as maltose, short-chain dextrins, and glucose, while glucoamylase (EC.3.2.1.3) can break the α-1,4 and α-1,6 glycosidic bonds of starch to convert starch into monosaccharides that can be used by microorganisms (Ji et al., 2021). These two GH play a key role in the utilization of raw materials by microorganisms during *huangjiu* fermentation. In this study, the enzymes of *S. rosea* A22 involved in starch, cellulose, and hemicellulose hydrolase include α-amylase (EC 3.2.1.1), Glucoamylase (EC 3.2.1.3), cellulase (EC 3.2.1.4), endo-1,3 (4)-beta-glucanase (EC 3.2.1.6), beta-xylosidase (EC 3.2.1.37) and alpha-mannosidase (EC 3.2.1.113) are also annotated in CAZys database (Supplementary Table S2). A total of 107 genes encoding related degradative enzymes were predicted in the *S. rosea* A22 genome, including 25 cellulose, 59 hemicellulose, and 8 starch degradation-related genes (Supplementary Table S2), 14 genes related to protease synthesis (Supplementary Table S3). These annotated genes suggest that *S. rosea* has great potential for degrading cellulose, hemicellulose, protein, and starch.

The issue of food safety has become of increasing global concern and recent developments have focused on the application of metabolomics for food safety control and food quality analysis (Li et al., 2021). A high content of biogenic amine in *huangjiu* can cause some damage to the body such as headache, impaired breathing, and blushing (Liu et al., 2021b). The strategy to reduce the content of biogenic amines in *huangjiu* was to screen microorganisms containing biogenic amine-degrading enzymes (Pištěková et al., 2020). The genome of *S. rosea* A22 contained 5 genes encoding biogenic amine degradation-related enzymes, primarily monoamine oxidase (EC 1.4.3.4) and primary amine oxidase (EC 1.4.3.21) (Supplementary Table S4), indicating that *S. rosea* had the potential for degrading biogenic amines in *huangjiu*.

### Stress resistance analysis of *Saccharopolyspora rosea* A22

**Figure 3:**
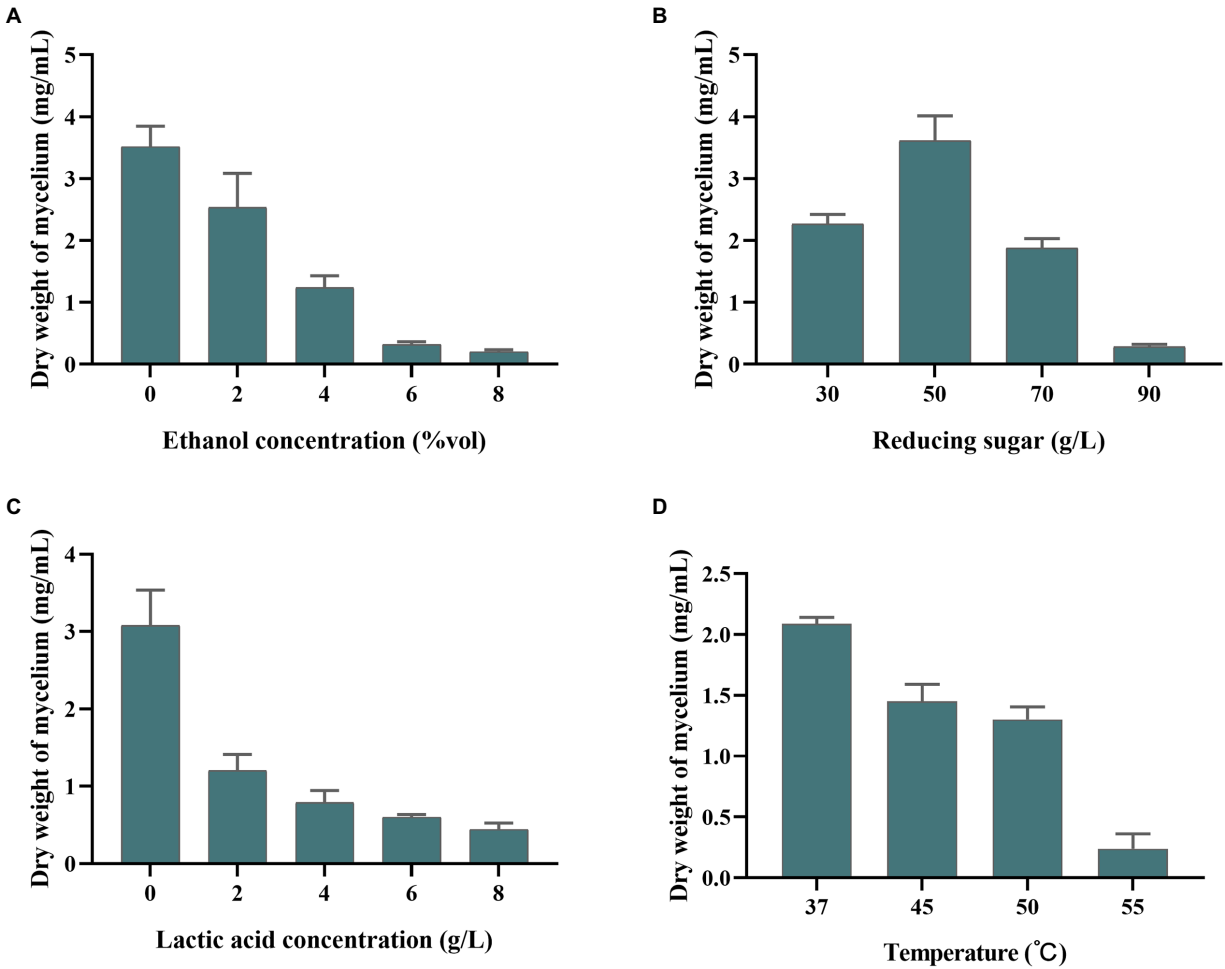
Tolerance of *S. rosea* A22 to different concentrations of ethanol **(A)**, reducing sugars **(B)**, lactic acid **(C)**, and different temperatures **(D)**.

*Saccharopolyspora* is crucial in the wheat *qu* (Wang et al., 2014; Zhang et al., 2022) and *huangjiu* (Liu et al., 2019). However, in the process of fermentation, *Saccharopolyspora* must survive in a complex stressful environment including ethanol, lactic acid, reducing sugar, and temperature. As shown in Figure 3A, the growth of *S. rosea* A22 was inhibited with the increase in ethanol concentration. The growth in 6–8%vol ethanol did not increase significantly, indicating that ethanol (>8%vol) inhibited the growth of *S. rosea* A22. The effect of reducing sugars on strain A22 was shown to be growth-promoting at low concentrations and growth-inhibiting at high concentrations (Figure 3B). The mycelial dry weight of strain A22 was 0.28 ± 0.03 mg/ml when the concentration of reducing sugar was 90 g/l, which indicated that the growth of *S. rosea* A22 had been inhibited. However, *huangjiu* fermentation is a simultaneous process of saccharification and fermentation, and the inhibition of microbial growth by high concentrations of reducing sugars diminishes as fermentation proceeds. Acid stress is also a challenge for *S. rosea* A22 (Figure 3C), and growth inhibition was most pronounced at lactate concentrations greater than 6 g/l. *S. rosea* A22 participated in the fermentation process of *huangjiu* by inoculating raw wheat *qu*, and the central temperature of the wheat *qu* fermentation process will reach 50–52°C (Xiao et al., 2017). The temperature tolerance test showed it can grow at 37–55°C (Figure 3D). Several stress-related genes of *S. rosea* A22 to temperature, osmotic stress, pH, oxidative stress, and drug resistance, were shown in Supplementary Table S5. The gene *grpE* was also thought to be a thermosensor of the *DnaK* system that changes its structure reversibly in response to thermal shock temperatures (Nakamura et al., 2010). Three genes of *grpE*, *hrcA*, and *IbpA*, encode heat shock proteins to respond to a sudden increase in temperature (Schulz and Schumann, 1996), and were identified in the *S. rosea* A22 genome. These annotated genes may be associated with heat tolerance (45–50°C) in *S. rosea* A22 (Figure 3D). The *S. rosea* A22 screened in this study was mainly used for the production of inoculated raw wheat *qu*, while the most commonly used microorganism in *huangjiu* inoculated with raw wheat *qu* is *Aspergillus flavus* (*A. flavus*) SU-16 (GCA_009856665.1) (Liu et al., 2020). However, due to the addition of cooked wheat *qu* inoculated with SU-16, the *huangjiu* exhibited a bitter and astringent taste, which seriously affected the drinking comfort of *huangjiu* (Lu et al., 2021). Therefore, there is an urgent need for a new wheat *qu* to replace the traditionally cooked wheat *qu*. In addition, only 1 heat tolerance gene, *HSPA*, was annotated to the genome of *Aspergillus flavus* (Supplementary Table S6). The acids and sugars simulated in this study were in a highly osmotic environment and the fact that strain A22 can survive may be related to encoding osmoregulatory proteins, such as the gene *opuC* encoding osmoprotectant binding protein. Notably, *S. rosea* A22 contained genes *pdtaR*, *pdtaS*, *opuC*, *engB,* and *choD* related to the osmotic environment (Supplementary Table S5). Genes *katE*, *cat*, *catB*, *srpA, katG*, and *nuoG* were associated with oxidative stress, which is a common challenge that most bacteria must overcome to survive (Gaupp et al., 2012). In addition, 12 salt tolerance genes including *kdpC*, *kdpB*, *kdpA*, *mnhE*, and *mrpE* were annotated in *S. rosea* A22 (Supplementary Table S5), which may be related to its good salt tolerance (Table 1). Only five salt tolerance genes have been annotated in the genome of *A. flavus* SU-16 (Supplementary Table S6) (Zhao et al., 2013). A total of 31 genes related to environmental tolerance were annotated in the genome of A22 (Supplementary Table S5), while only 16 genes related to environmental tolerance were annotated in the genome of *Aspergillus flavus* (Supplementary Table S6), indicating that *S. rosea* A22 is more likely to exert its function than *A. flavus* SU-16 in the harsh environment of *huangjiu* fermentation.

### Potential for production of secondary metabolites

**Figure 4:**
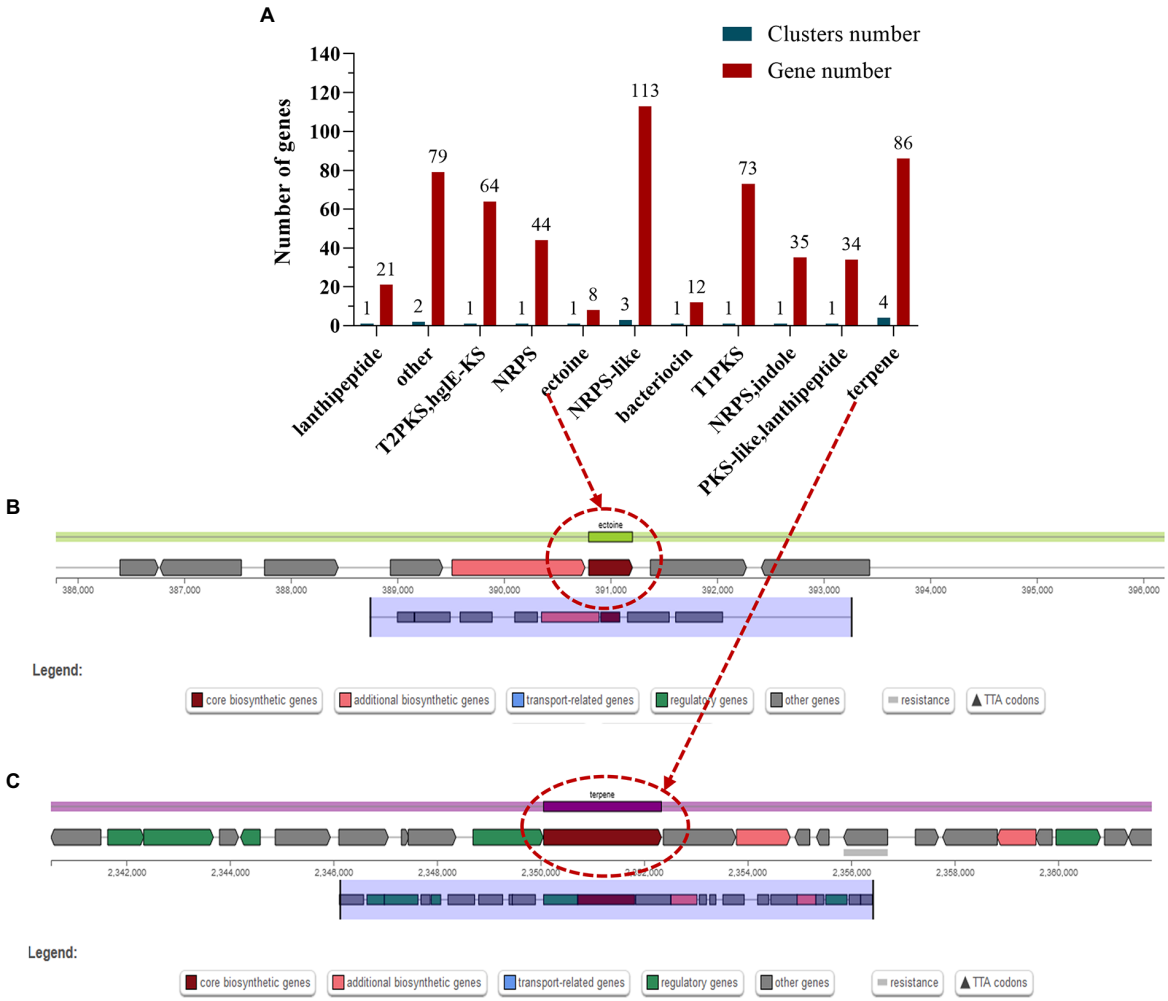
Classification statistics **(A)** of the secondary metabolite biosynthesis gene clusters in the *S. rosea* A22 genome and the chromosomal location of the key secondary metabolites ectoine **(B)** and terpene **(C)**.

Secondary metabolites are biologically active compounds synthesized by microorganisms using primary metabolites as precursors, which may be involved in the physiological processes of the bacterium itself and may also provide defense and resistance to external stressful environments (Liu et al., 2022). The most common secondary metabolic gene clusters are type I, II, and III polyketides synthase (PKS) and non-ribosomal peptides synthase (NRPS) (Medema et al., 2011). Prediction of the genome of *S. rosea* A22 using antiSMASH-4.0.2 revealed a total of 17 biosynthetic gene clusters, and 3 gene clusters comprising 113 genes were annotated in the NRPS-like classification (Figure 4A). Interestingly, the ectoine in cluster 1 is 100% similar to the ectoine cluster of *Streptomyces anulatus* (Supplementary Table S7). Ectoine has high water solubility, can improve the balance ability of osmotic pressure, stabilize and protect the enzyme activity in bacteria, and is a stress-resistant protective agent for microorganisms under the conditions of high salt, drying, and freezing (Pastor et al., 2010). There are eight genes associated with ectoine synthesis, with ectoine synthase being the key gene for ectoine synthesis (Figure 4B), and the *ectA*, *ectB*, *ectC*, and *ectD* genes associated with ectoine synthesis and catabolism (Supplementary Table S8). In addition, terpene synthase key gene *cyc*2 present in cluster 6 (Figure 4C and Supplementary Table S8), showed 100% similarity to the gene cluster that produces geosmin from *Saccharopolyspora erythraea* (Liu et al., 2013), indicating that *S. rosea* A22 has the potential to produce geosmin.

### Genome-scale metabolic modeling of strain *Saccharopolyspora rosea*

#### General model properties

**Figure 5:**
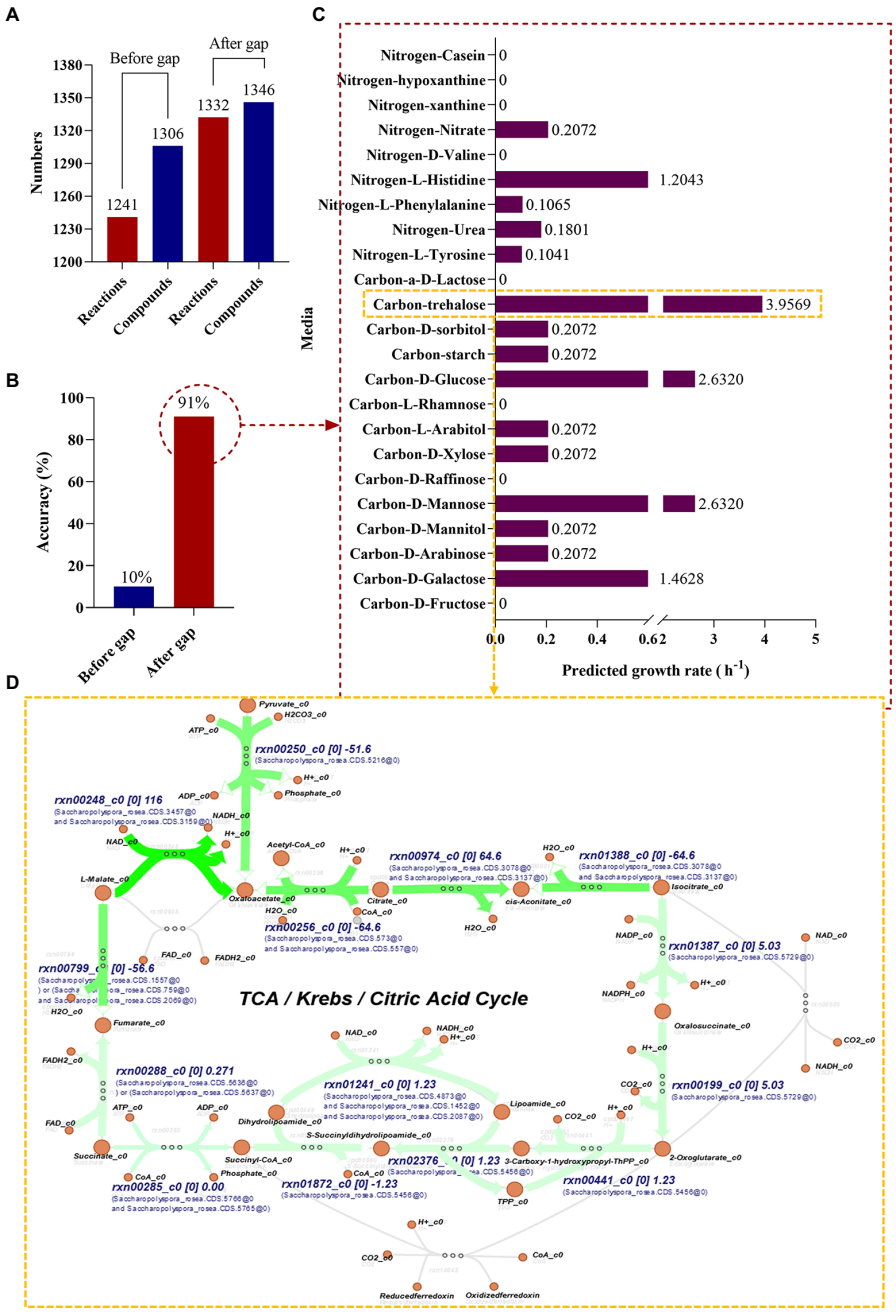
Genome-scale metabolic model construction and growth prediction of *S. rosea* A22 in different media. **(A)** Number of reactions and compounds included in the model. **(B)** The accuracy of prediction based on phenotypic data. **(C)** Flow balance analysis on different media. **(D)** Visualization of flux distribution of trehalose as a carbon source in the TCA cycle pathway.

In this study, the genome was annotated using the RAST (see text footnote 4) and the *S. rosea* A22 subsystem distribution was obtained (Figure 2E), mainly focusing on amino acids and derivatives (19.81%), carbohydrates (19.81%), cofactors, vitamins and other (10.87%), protein metabolism (10.29%). A genome-wide metabolic model of *S. rosea* A22 was constructed based on KBase. The final sketch metabolic model contained a total of 1,310 genes, 1,306 metabolites, 1,241 reactions, and 2 compartments for cytosol and extracellular (Figure 5A and Supplementary Table S10).

#### Manual curation

Bergey’s manual of determinative bacteriology demonstrates that *S. rosea* can utilize multiple carbon and nitrogen sources. We manually designed 23 media (Supplementary Table S11) with individual carbon or nitrogen sources to gap (artificially added metabolites and reactions) the metabolic model of *S. rosea* A22. The final refined model of *S. rosea* was obtained, including 1,310 genes, 1,346 metabolites, 1,332 reactions, cytosol, and extracellular 2 compartments, 40 metabolites, and 90 reactions were added. The “check model mass balance” function in KBase was used to check the mass-charge balance after gapping, and the results showed that the mass-charge balance on both sides of the reaction. The refined model was named *i*SR1310 following the convention *i* for *in silico*, combining the species name (*S. rosea*) abbreviations and genes (1310) (Enuh and Aytar Çelik, 2022).

#### Simulate growth on phenotype data

After the construction of the model *i*SR1310, we validated the reliability of the model through predictive *in silico* and “wet experiments” *in vitro*. The carbon sources utilized by *S. rosea* are galactose, arabinose, mannose, mannitol, arabinitol, xylose, glucose, starch, and sorbitol, and the nitrogen sources utilized are mainly tyrosine, urea, phenylalanine, histidine, hypoxanthine, and casein (Kim and Goodfellow, 2015). Then, we used the “wet experiment” as the phenotype data to evaluate the prediction accuracy of the model before and after the gap through the function of “simulate growth on phenotype data” in KBase, and it was obvious that the prediction accuracy of the model after the gap reached 91% (Figure 5B). In addition, it was found that the model predictions and experimental results were consistent for the utilization of carbon and nitrogen sources, except for casein and hypoxanthine (Table 3). The above studies show that the model *i*SR1310 we constructed had high accuracy in predicting the growth of *S. rosea* on different media. Flow balance analysis (FBA) is to investigate simulations interactively by modifying parameters and receiving immediate feedback (Rowe et al., 2018). To investigate the ability of *S. rosea* to utilize carbon and nitrogen sources, we used KBase to simulate the growth of the model *i*SR1310 on different media (Figure 5C). The metabolic flux of *S. rosea* was higher in the medium with mannose, glucose, and trehalose as the sole carbon source and histidine as the sole nitrogen source. Obviously, FBA predicted that the model *i*SR1310 had the greatest growth rate (3.9569 (h^−1^)) when trehalose was used as the sole carbon source to simulate growth. Therefore, we used Escher maps to visualize the flux distributions for trehalose as a carbon source in the TCA cycle pathway (Figure 5D). To illustrate Figure 5D more clearly, I take NADH synthesis as an example, “rxn00248” represents the reactions involved, “c0” represents the compartments in the cytosol, and “116” represents the interactive metabolic flux, and “Saccharopolyspora_rosea.CDS.3475” represents the gene. Obviously, the synthetic intermediate metabolites ADP, NADH, acetyl coenzyme A, fumarate, oxaloacetate, and H_2_O have a higher metabolic flow than the other metabolites. Meanwhile, we measured the growth rate of A22 with trehalose as the sole carbon source, and found that the error between the experimental value and the predicted value was 5.73% (3.75 vs. 3.9569) (Supplementary Figure S3), which was less than 10% (Wang et al., 2015), indicating that *i*SR1310 can accurately reflect the growth on the medium state.

**Table 3:** Predicting the growth of model *i*SR1310 on different media based on phenotypic data.

Growth condition	Observed normal growth	Simulated growth	Prediction class
Carbon-D-fructose	0	0	CN
Carbon-D-galactose	1	1	CP
Carbon-D-arabinose	1	1	CP
Carbon-D-trehalose	1	1	CP
Carbon-D-mannitol	1	1	CP
Carbon-D-mannose	1	1	CP
Carbon-D-raffinose	0	0	CN
Carbon-D-xylose	1	1	CP
Carbon-L-arabitol	1	1	CP
Carbon-L-rhamnose	0	0	CN
Carbon-D-glucose	1	1	CP
Carbon-a-D-lactose	0	0	CP
Carbon-starch	1	1	CP
Carbon-sorbitol	1	1	CP
Nitrogen-L-tyrosine	1	1	CP
Nitrogen-urea	1	1	CP
Nitrogen-L-phenylalanine	1	1	CP
Nitrogen-L-histidine	1	1	CP
Nitrogen-D-valine	0	0	CN
Nitrogen-xanthine	0	0	CN
Nitrogen-hypoxanthine	1	0	FN
Nitrogen-casein	1	0	FN
Nitrogen-nitrate	1	1	CP

1, growth; 0, no growth; CP, correct positions; CN, correct negatives; FP, false positions: FN, false negatives.

#### Application of *Saccharopolyspora rosea* A22 to *huangjiu* fermentation

**Figure 6:**
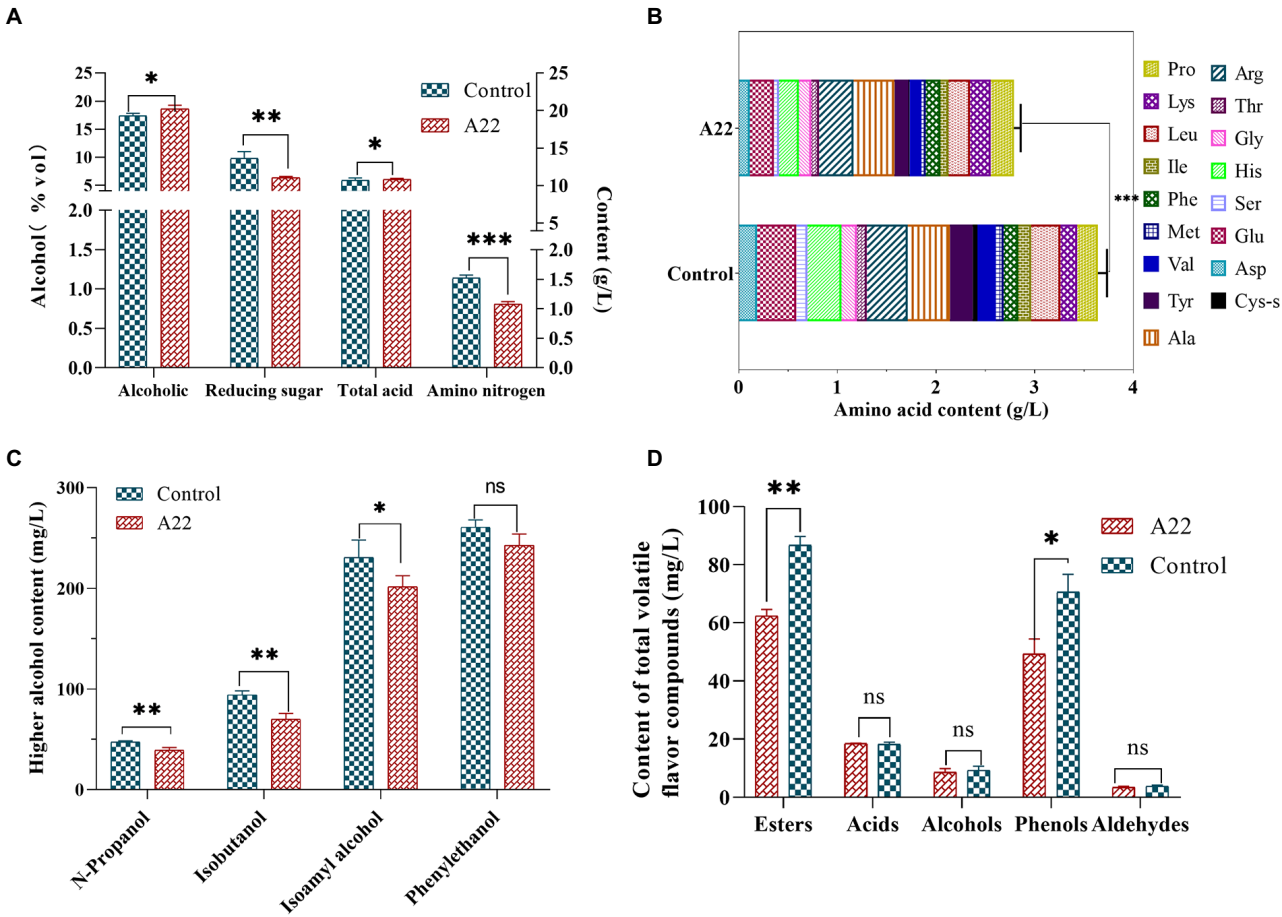
Physicochemical indexes **(A)**, content of free amino acid **(B)**, higher alcohol **(C),** and total flavor substance **(D)** of *huangjiu*. ns, not significant; ^*^p < 0.05 (moderately significant); ^**^p < 0.01 (significant); and ^***^p < 0.001 (highly significant).

To further verify the potential of *S. rosea* A22 for *huangjiu* fermentation, *S. rosea* A22 was inoculated into raw wheat *qu* for *huangjiu* fermentation experiments. The ethanol concentration of the A22 group was significantly higher than that of the control group (18.60%vol vs. 17.40%vol), while the reducing sugar content was significantly lower than that of the control group (6.36 g/l vs. 9.82 g/l), which indicated that *S. rosea* A22 provided more enzymes for the hydrolysis of starch to facilitate the growth of yeast for ethanol production (Sayed et al., 2020). However, the content of amino nitrogen in the experimental group was significantly lower than that in the control group (0.81 g/l vs. 1.37 g/l), indicating that A22 was less capable of producing protease and peptidase and did not completely hydrolyze the proteins in the *huangjiu* fermentation mash to amino nitrogen (Figure 6A). Free amino acids are not only the precursors for the synthesis of esters and alcohols in *Saccharomyces cerevisiae*, but also indicators of the quality of *huangjiu* (Gambetta et al., 2017). The content of total free amino acids in the control group was significantly higher than that treated groups (3.64 g/l vs. 2.78 g/l) (Figure 6B), and the main reason is that the wheat *qu* in the control group contains *Aspergillus flavus* with high protease production. The levels of bitter amino acids including His, Arg, Val, Phe, Ile, Leu, and Lys were lower in the A22 group than in the control group (1.33 g/l vs. 1.63 g/l), indicating it will be a potential substitute for wheat *qu* in mechanized *huangjiu*.

Higher alcohols, as the key flavor compounds in *huangjiu*, can enrich the full, round and harmonious taste of *huangjiu*, but high levels of higher alcohols will lead to adverse symptoms such as headache and nausea in consumers (Chen et al., 2013). In this study, the content of total higher alcohols in the control group was higher than that in the A22 group (633 g/l vs. 553 g/l, decreased by 12.64%), and isobutanol and isoamyl alcohol decreased by 25 and 13%, respectively, compared with the control group (Figure 6C). These results show that the application of *S. rosea* A22 in the fermentation of *huangjiu* can improve post-drinking comfort. In addition, the contents of esters (87 mg/l vs. 62 mg/l) and phenols (71 mg/l vs. 49 mg/l) in the control group were significantly higher than those in the A22 group (Figure 6D). Guaiacol and 4-vinyl guaiacol constituted the main phenols in this study, and contributed to the clove, spicy, and smoky odor. Previous studies have shown that guaiacol and 4-vinyl guaiacol are rich in wheat koji, suggesting that guaiacol and 4-vinyl guaiacol in *huangjiu* mainly come from wheat *qu* (Chen and Xu, 2013). GC-O indicated that acetic acid was probably the most important volatile acid in *huangjiu* (Yu et al., 2019). Isovallardehyde and furfural constitute the main aldehydes (Supplementary Table S9). In this study, there was no significant difference in aldehydes, acids, and alcohols between the control group and the experimental group.

## Conclusion

In this study, we sequenced and described the first whole genome of *S. rosea* A22 as 6,562,638 bp with an average GC content of 71.71%, and 6,118 CDS. Genomic functional analysis showed that *S. rosea* has the potential to produce protease, amylase, cellulase, and hemicellulase, indicating that it can enhance the utilization of raw materials in fermented foods. *S. rosea* A22 had been shown to have good tolerance to temperature, acid, ethanol, and sugar in extreme environments. Analysis of tolerance mechanisms indicated that heat tolerance may be associated with regulated heat proteins genes *grpE*, *hrcA*, and *IbpA*, and acid, sugar, and ethanol tolerance may be associated with regulated osmotic proteins genes *pdtaR*, *pdtaS*, *opuC*, *engB,* and *choD*. Additionally, we firstly constructed and reported the first genome-scale metabolic model of *S. rosea* A22 named *i*SR1310, which can predict the growth ability of *S. rosea* on different media with 91% accuracy. Finally, *S. rosea* A22 was applied to *huangjiu* fermentation by inoculating raw wheat *qu*, and the results showed that the ethanol content of the A22 group was significantly higher than that of the control group (18.6%vol vs. 17.4%vol), while the reducing sugar content (6.36 g/l vs. 9.82 g/l) and amino nitrogen (0.81 g/l vs. 1.37 g/l) were significantly lower than that of the control group, respectively. Also, the results showed that the total higher alcohol content was reduced by 12.64% compared with the control group, which indicated that *S. rosea* A22 can improve the comfort of *huangjiu*, and has great potential for application in *huangjiu*.

## Data availability statement

The 16S rRNA sequence of *Saccharopolyspora rosea* A22 presented in the study was deposited in the NCBI repository, accession number OP218373. The whole genome sequence of *Saccharopolyspora rosea* A22 was deposited in the NCBI repository, accession number PRJNA869361.

## Author contributions

DM: conceptualization, methodology, formal analysis, investigation, visualization, and writing – original draft. SL, XH, YX, BQ, and LW: supervision, writing – review, and editing. MN: data curation, writing – review, and editing. JM: funding acquisition, supervision, writing – review, and editing. All authors contributed to the article and approved the submitted version.

## Funding

This research was funded by the National Natural Science Foundation of China (32072205 and 22138004) and the first phase of the connotation construction of the 14th Five-Year Plan of Tibetan medicine (2021ZYYGH008).

## Conflict of interest

SL, XH, YX, BQ, LW, and JM were employed by Zhejiang Guyuelongshan Shaoxing Wine Co., Ltd.

The remaining authors declare that the research was conducted in the absence of any commercial or financial relationships that could be construed as a potential conflict of interest.

## Publisher’s note

All claims expressed in this article are solely those of the authors and do not necessarily represent those of their affiliated organizations, or those of the publisher, the editors and the reviewers. Any product that may be evaluated in this article, or claim that may be made by its manufacturer, is not guaranteed or endorsed by the publisher.

## Supplementary material

The Supplementary material for this article can be found online at: https://www.frontiersin.org/articles/10.3389/fmicb.2022.995978/full#supplementary-material

## Footnotes

^1^ www.bacterio.net/saccharopolyspora.html

^2^ https://www.ncbi.nlm.nih.gov/genome/term=*Saccharopolyspora*

^3^ http://kbase.us/

^4^ https://rast.nmpdr.org/
