# Supplementary material for: Complete genome sequence, metabolic model construction, and huangjiu application of Saccharopolyspora rosea A22, a thermophilic, high amylase and glucoamylase actinomycetes
Source: Front Microbiol. 2022 Sep 28;13:995978. doi: 10.3389/fmicb.2022.995978 (PMC9554608; doi:10.3389/fmicb.2022.995978)
Supplement: Supplementary file 3 [file Data_Sheet_1.docx]

**Complete genome sequence, metabolic model construction, and *huangjiu* application of *Saccharopolyspora rosea* A22, a thermophilic, high amylase and glucoamylase actinomycetes**

Run title: Analysis of *Saccharopolyspora rosea*

Donglin Ma^1^, Shuangping Liu^1, 2, 3*^, Xiao Han^1, 2, 3^, Mujia Nan^4^, Yuezheng Xu^3^, Bin Qian^3^, Lan Wang^3^ and Jian Mao^1, 2, 3*^


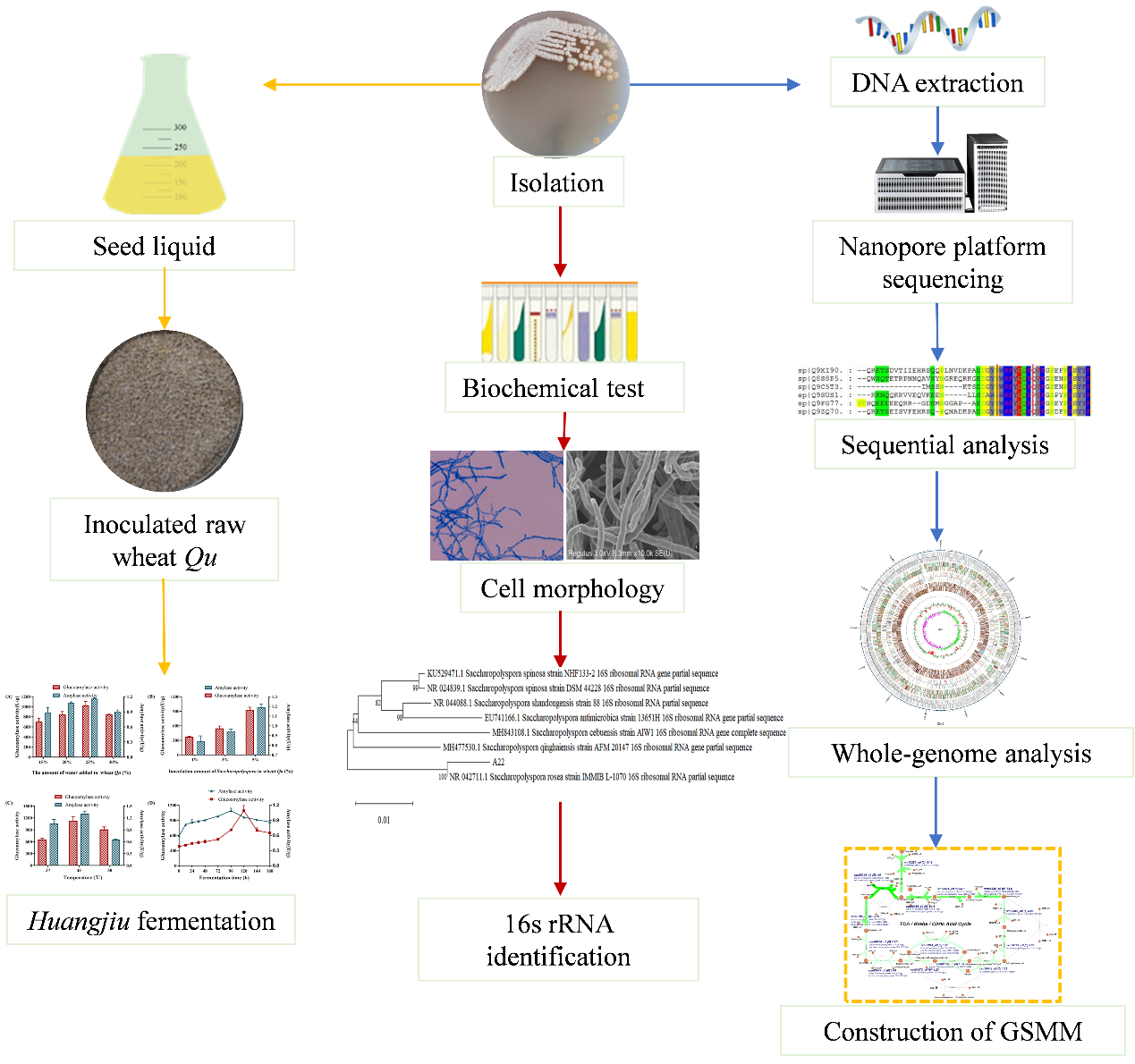


**Figure S1.** The flow chart of the entire work


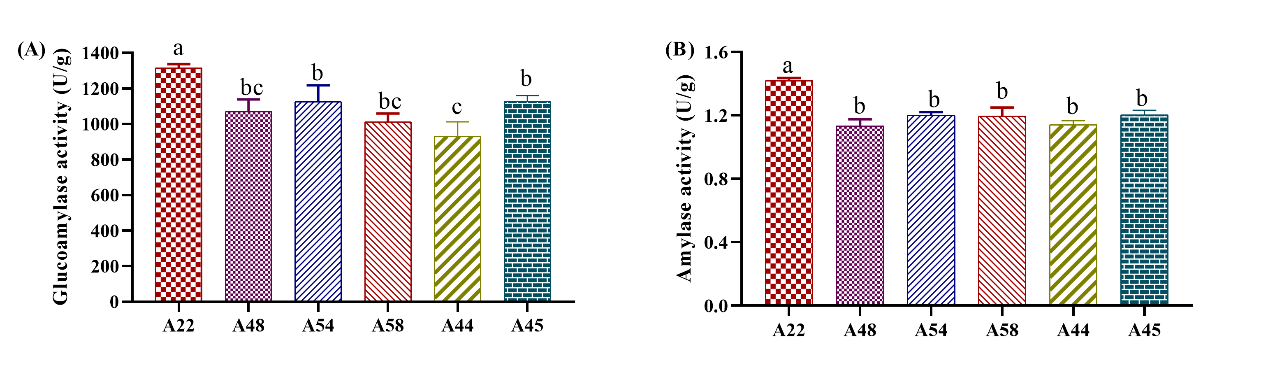


**Figure S2.** Glucoamylase (A) and amylase (B) of wheat *Qu* inoculated with different strains





**Figure S3.** Experimental determination of changes in biomass (A) and growth rate (B) of *S. rosea* A22 with trehalose as the sole carbon source

**Table S1** The results of amylase production by different strains

| Strains | Hydrolysis circle（H, cm） | Colony diameter  (C, cm) | H/C | Strains | Hydrolysis circle（H, cm） | Colony diameter  (C, cm) | H/C |
| --- | --- | --- | --- | --- | --- | --- | --- |
| Control | 5.80 | 0.31 | 18.71 | A-38 | 6.00 | 0.31 | 19.35 |
| A-1 | 5.50 | 0.32 | 17.19 | A-39 | 5.80 | 0.31 | 18.71 |
| A-2 | 5.80 | 0.31 | 18.71 | A-40 | 6.20 | 0.30 | 20.67 |
| A-3 | 6.21 | 0.32 | 19.41 | A-41 | 6.10 | 0.31 | 19.68 |
| A-4 | 5.51 | 0.31 | 17.77 | A-42 | 6.40 | 0.30 | 21.33 |
| A-5 | 6.10 | 0.32 | 19.06 | A-43 | 5.40 | 0.31 | 17.42 |
| A-6 | 5.90 | 0.30 | 19.67 | A-44 | 7.10 | 0.31 | 22.90 |
| A-7 | 5.80 | 0.31 | 18.71 | A-45 | 7.20 | 0.30 | 24.00 |
| A-8 | 6.00 | 0.30 | 20.00 | A-46 | 6.80 | 0.30 | 22.67 |
| A-9 | 5.60 | 0.33 | 16.97 | A-47 | 6.30 | 0.30 | 21.00 |
| A-10 | 5.90 | 0.32 | 18.44 | A-48 | 7.00 | 0.31 | 22.58 |
| A-11 | 6.00 | 0.31 | 19.35 | A-49 | 6.90 | 0.30 | 23.00 |
| A-12 | 5.51 | 0.31 | 17.77 | A-50 | 5.80 | 0.30 | 19.33 |
| A-13 | 5.30 | 0.32 | 16.56 | A-51 | 6.50 | 0.31 | 20.97 |
| A-14 | 5.90 | 0.30 | 19.67 | A-52 | 6.90 | 0.30 | 23.00 |
| A-15 | 6.00 | 0.31 | 19.35 | A-53 | 5.80 | 0.30 | 19.33 |
| A-16 | 6.00 | 0.31 | 19.35 | A-54 | 7.30 | 0.31 | 23.55 |
| A-17 | 5.80 | 0.32 | 18.13 | A-55 | 6.90 | 0.30 | 23.00 |
| A-18 | 6.10 | 0.31 | 19.68 | A-56 | 6.30 | 0.31 | 20.32 |
| A-19 | 6.50 | 0.30 | 21.67 | A-57 | 5.90 | 0.31 | 19.03 |
| A-20 | 6.21 | 0.32 | 19.41 | A-58 | 7.30 | 0.31 | 23.55 |
| A-21 | 7.51 | 0.31 | 24.23 | A-59 | 7.00 | 0.30 | 23.33 |
| A-22 | 7.50 | 0.28 | 26.79 | A-60 | 6.80 | 0.31 | 21.94 |
| A-23 | 6.50 | 0.30 | 21.67 | A-61 | 6.60 | 0.29 | 22.76 |
| A-24 | 6.00 | 0.31 | 19.35 | A-62 | 5.90 | 0.30 | 19.67 |
| A-25 | 7.10 | 0.30 | 23.67 | A-63 | 6.00 | 0.31 | 19.35 |
| A-26 | 7.00 | 0.33 | 21.21 | A-64 | 5.50 | 0.30 | 18.33 |
| A-27 | 7.00 | 0.32 | 21.88 | A-65 | 5.70 | 0.31 | 18.39 |
| A-28 | 6.50 | 0.31 | 20.97 | A-66 | 5.80 | 0.30 | 19.33 |
| A-29 | 5.51 | 0.31 | 17.77 | A-67 | 5.50 | 0.30 | 18.33 |
| A-30 | 6.60 | 0.32 | 20.63 | A-68 | 7.00 | 0.31 | 22.58 |
| A-31 | 6.50 | 0.30 | 21.67 | A-69 | 6.90 | 0.31 | 22.26 |
| A-32 | 6.00 | 0.31 | 19.35 | A-70 | 5.80 | 0.31 | 18.71 |
| A-33 | 6.00 | 0.31 | 19.35 | A-71 | 6.00 | 0.31 | 19.35 |
| A-34 | 6.50 | 0.32 | 20.31 | A-72 | 5.60 | 0.30 | 18.67 |
| A-35 | 6.10 | 0.31 | 19.68 | A-73 | 6.30 | 0.31 | 20.32 |
| A-36 | 6.50 | 0.30 | 21.67 | A-74 | 6.90 | 0.31 | 22.26 |
| A-37 | 6.40 | 0.31 | 20.65 | A-75 | 5.40 | 0.31 | 17.42 |

**Table S2** Starch-, cellulose- and hemicellulose-related genes annotated in the *S. rosea* A22 genome

| **Category** | **Predicted function** | **EC number** | **CAZyme families** | **Protein ID** |
| --- | --- | --- | --- | --- |
| Cellulose-degrading enzymes | alpha-glucosidase | 3.2.1.20 | GH4 | A22_GM001349 |
|  |  | 3.2.1.107 | GH65 | A22_GM003623 |
|  |  | 3.2.1.107 | GH65 | A22_GM000101 |
|  |  | 3.2.1.20 | GH13 | A22_GM002487 |
|  | beta-glucosidase | 3.2.1.21 | GH2 | A22_GM003979 |
|  |  | 3.2.1.21 | GH3 | A22_GM002304 |
|  |  | 3.2.1.21 | GH1 | A22_GM002448 |
|  |  | 3.2.1.21 | GH3 | A22_GM000268 |
|  |  | 3.2.1.21 | GH2 | A22_GM004614 |
|  |  | 3.2.1.21 | GH1 | A22_GM005665 |
|  |  | 3.2.1.21 | GH2 | A22_GM005531 |
|  | lytic cellulose monooxygenase (C1-hydroxylating) | 1.14.99.54 | AA10 | A22_GM003820 |
|  |  | 1.14.99.54 | AA10 | A22_GM000074 |
|  |  | 1.14.99.54 | AA10 | A22_GM005196 |
|  | Lytic polysaccharide monooxygenases (LPMOs) |  | AA10 | A22_GM003820 |
|  |  |  | AA10 | A22_GM000074 |
|  |  |  | AA10 | A22_GM005196 |
|  | xyloglucanase |  | GH16 | A22_GM003462 |
|  |  |  | GH16 | A22_GM000330 |
|  | β-N-Acetylhexosaminidase | 3.2.1.52 | GH84 | A22_GM005840 |
|  |  |  | GH3 | A22_GM002304 |
|  |  |  | GH3 | A22_GM000268 |
|  |  |  | GH84 | A22_GM000407 |
|  |  |  | GH20 | A22_GM001816 |
|  |  |  | GH20 | A22_GM005258 |
| **Hemicellulose-degrading enzymes** | Xylan 1,4-β-xylosidase | 3.2.1.37 | GH2 | A22_GM003979 |
|  |  |  | GH3 | A22_GM002304 |
|  |  |  | GH1 | A22_GM002448 |
|  |  |  | GH3 | A22_GM000268 |
|  |  |  | GH2 | A22_GM004614 |
|  |  |  | GH1 | A22_GM005665 |
|  |  |  | GH2 | A22_GM005531 |
|  | α-L-Arabinofuranosidase | 3.2.1.55 | GH2 | A22_GM003979 |
|  |  | 3.2.1.55 | GH3 | A22_GM002304 |
|  |  |  | GH3 | A22_GM000268 |
|  |  |  | GH2 | A22_GM004614 |
|  |  |  | GH2 | A22_GM005531 |
|  | Endo-1,3-beta-D-glucosidase | 3.2.1.39 | GH64 | A22_GM004508 |
|  |  |  | GH55 | A22_GM002056 |
|  |  |  | GH16 | A22_GM003462 |
|  |  |  | GH16 | A22_GM000330 |
|  | Endo-1,3(4)-β-glucanase | 3.2.1.6 | GH1 | A22_GM002448 |
|  |  |  | GH4 | A22_GM001349 |
|  |  |  | GH13 | A22_GM002487 |
|  |  |  | GH1 | A22_GM005665 |
|  |  |  | GH16 | A22_GM003462 |
|  |  |  | GH28 | A22_GM004561 |
|  |  |  | GH16 | A22_GM000330 |
|  |  |  | GH28 | A22_GM002777 |
|  | Lichenase | 3.2.1.73 | GH16 | A22_GM003462 |
|  |  |  | GH16 | A22_GM000330 |
|  | α-Galactosidase | 3.2.1.22 | GH4 | A22_GM001349 |
|  |  |  | GH27 | A22_GM005921 |
|  | β-Mannosidase | 3.2.1.25 | GH2 | A22_GM003979 |
|  |  |  | GH1 | A22_GM002448 |
|  |  |  | GH2 | A22_GM004614 |
|  |  |  | GH1 | A22_GM005665 |
|  |  |  | GH2 | A22_GM005531 |
|  | β-Glucuronidase | 3.2.1.31 | GH2 | A22_GM003979 |
|  |  |  | GH1 | A22_GM002448 |
|  |  |  | GH2 | A22_GM004614 |
|  |  |  | GH1 | A22_GM005665 |
|  |  |  | GH2 | A22_GM005531 |
|  | β-Galactosidase | 3.2.1.23 | GH2 | A22_GM003979 |
|  |  |  | GH1 | A22_GM002448 |
|  |  |  | GH35 | A22_GM000231 |
|  |  |  | GH2 | A22_GM004614 |
|  |  |  | GH1 | A22_GM005665 |
|  |  |  | GH2 | A22_GM005531 |
|  | Acetylxylan esterase | 3.1.1.72 | CE12 | A22_GM004564 |
|  |  |  | CE4 | A22_GM003996 |
|  | 1,3-β-Glucosidase | 3.2.1.58 | GH55 | A22_GM002056 |
|  | α-Glucuronidase | 3.2.1.139 | GH4 | A22_GM001349 |
|  | Polygalacturonase | 3.2.1.15 | GH2 | A22_GM003979 |
|  |  |  | GH3 | A22_GM002304 |
|  |  |  | GH3 | A22_GM000268 |
|  |  |  | GH2 | A22_GM004614 |
|  |  |  | GH16 | A22_GM003462 |
|  |  |  | GH2 | A22_GM005531 |
|  |  |  | GH28 | A22_GM004561 |
|  |  |  | GH16 | A22_GM000330 |
|  |  |  | GH28 | A22_GM002777 |
|  | Rhamnogalacturonan hydrolase | 3.2.1.171 | GH28 | A22_GM004561 |
|  |  |  | GH28 | A22_GM002277 |
| **Starch-degrading enzymes** | alpha-amylase | 3.2.1.1 | GH13 | A22_GM002487 |
|  | glucoamylase | 3.2.1.3 | GH15 | A22_GM000351 |
|  |  |  | GH15 | A22_GM003584 |
|  |  |  | GH15 | A22_GM004203 |
|  | isoamylase | 3.2.1.68 | GH13 | A22_GM002487 |
|  | maltogenic amylase | 3.2.1.133 | GH13 | A22_GM002487 |
|  | cyclomaltodextrinase | 3.2.1.54 | GH13 | A22_GM002487 |
|  | neopullulanase | 3.2.1.135 | GH13 | A22_GM002487 |

**Table S3** Protease-related genes annotated in the *S. rosea* A22 genome

| Gene_id | Ko_name | Ko_definition | Ko_EC |
| --- | --- | --- | --- |
| A22_GM000297 | *dacB* | serine-type D-Ala-D-Ala carboxypeptidase/endopeptidase (penicillin-binding protein 4) | 3.4.16.4 3.4.21.- |
| A22_GM000613 | *pepD* | putative serine protease PepD | 3.4.21.- |
| A22_GM001047 | *PREP* | prolyl oligopeptidase | 3.4.21.26 |
| A22_GM001079 | *clpP, CLPP* | ATP-dependent Clp protease, protease subunit | 3.4.21.92 |
| A22_GM001080 | *clpP, CLPP* | ATP-dependent Clp protease, protease subunit | 3.4.21.92 |
| A22_GM001191 | *clpP, CLPP* | ATP-dependent Clp protease, protease subunit | 3.4.21.92 |
| A22_GM001192 | *clpP, CLPP* | ATP-dependent Clp protease, protease subunit | 3.4.21.92 |
| A22_GM004695 | *dacB* | serine-type D-Ala-D-Ala carboxypeptidase/endopeptidase (penicillin-binding protein 4) | 3.4.16.4 3.4.21.- |
| A22_GM004937 | *lepB* | signal peptidase I | 3.4.21.89 |
| A22_GM005632 | *mycP* | membrane-anchored mycosin MYCP | 3.4.21.- |
| A22_GM003220 | *hyaD, hybD* | hydrogenase maturation protease | 3.4.23.- |
| A22_GM004514 | *pilD, pppA* | leader peptidase (prepilin peptidase) / N-methyltransferase | 3.4.23.43 2.1.1.- |
| A22_GM004763 | *lspA* | signal peptidase II | 3.4.23.36 |
| A22_GM003528 | *E2.8.3.5B, scoB* | 3-oxoacid CoA-transferase subunit B | 2.8.3.5 |
| A22_GM003529 | *E2.8.3.5A, scoA* | 3-oxoacid CoA-transferase subunit A | 2.8.3.5 |

**Table S4** Biogenic amine degrading enzyme genes in the *S. rosea* A22 genome

| Gene_id | Ko_name | Ko_defi | Ko_EC |
| --- | --- | --- | --- |
| A22_GM001965 | *MAO, aofH* | monoamine oxidase | 1.4.3.4 |
| A22_GM003432 | *MAO, aofH* | monoamine oxidase | 1.4.3.4 |
| A22_GM003513 | *MAO, aofH* | monoamine oxidase | 1.4.3.4 |
| A22_GM003829 | *MAO, aofH* | monoamine oxidase | 1.4.3.4 |
| A22_GM001685 | *AOC3, AOC2* | Primary amine-oxidase | 1.4.3.21 |

**Table S5** Stress-related genes of *S. rosea* A22

| Stress | Gene id | Gene | Functional_description |
| --- | --- | --- | --- |
| Temperature | A22_GM001163 | *hrcA* | Transcriptional regulator of heat shock response |
|  | A22_GM001789 | *grpE* | Molecular chaperone GrpE (heat shock protein HSP-70) |
|  | A22_GM005978 |  |  |
|  | A22_GM006038 |  |  |
|  | A22_GM002595 | *IbpA* | Small heat shock protein IbpA, HSP20 family |
|  | A22_GM002882 |  |  |
| Osmotic stress | A22_GM001632 | *pdtaR* | Two-component response regulator, AmiR/NasT family, consists of REC and RNA-binding antiterminator (ANTAR) domains |
|  | A22_GM005275 | *pdtaS* | Two-component sensor histidine kinase, HisKA and HATPase domains |
|  | A22_GM000047 | *-* | Choline-glycine betaine transporter |
|  | A22_GM000051 | *opuC* | Periplasmic glycine betaine/choline-binding (lipo)protein of an ABC-type transport system (osmoprotectant binding protein) |
|  | A22_GM005260 |  |  |
|  | A22_GM001676 | *engB* | Choline dehydrogenase or related flavoprotein |
|  | A22_GM005567 | *choD* | Choline dehydrogenase or related flavoprotein |
| pH | A22_GM005567 | *BetA* | Uncharacterized membrane permease YidK, sodium, solute symporter family |
| Oxidative stress | A22_GM000214 | *katE, cat, catB, srpA;* | Catalase |
|  | A22_GM003946 |  |  |
|  | A22_GM002153 | *katG* | Catalase (peroxidase I) |
|  | A22_GM005727 | *nuoG* | NADH dehydrogenase/NADH, ubiquinone oxidoreductase 75 kD subunit (chain G) |
| Drug resitance | A22_GM003960 | *penP* | Beta-lactamase class A |
| Salt-tolerance | A22_GM004980 | *kdpC* | K^+^-transporting ATPase ATPase C chain |
|  | A22_GM004981 | *kdpB* | K^+^-transporting ATPase ATPase B chain |
|  | A22_GM004982 | *kdpA* | K+-transporting ATPase ATPase A chain |
|  | A22_GM005385 | *mnhG, mrpG* | multicomponent Na^+^:H^+^ antiporter subunit G |
|  | A22_GM005386 | *mnhF, mrpF* | multicomponent Na^+^:H^+^ antiporter subunit F |
|  | A22_GM005387 | *mnhE, mrpE* | multicomponent Na^+^:H^+^ antiporter subunit E |
|  | A22_GM005388 | *mnhD, mrpD* | multicomponent Na^+^:H^+^ antiporter subunit D |
|  | A22_GM005389 | *mnhC, mrpC* | multicomponent Na^+^:H^+^ antiporter subunit C |
|  | A22_GM005390 | *mnhB, mrpB* | multicomponent Na^+^:H^+^ antiporter subunit B |
|  | A22_GM005391 | *mnhA, mrpA* | multicomponent Na^+^:H^+^ antiporter subunit A |
|  | A22_GM000258 | *nhaA* | Na^+^:H^+^ antiporter, NhaA family |
|  | A22_GM003044 | *TC.BASS* | bile acid: Na^+^ symporter, BASS family |

**Table S6** Stress-related genes of *Aspergillus flavus* SU-16

| Gene id | KO | Definition |
| --- | --- | --- |
| **Salt-tolerance related genes** |  |  |
| evm. model. Chromosome3.521 | K01536 | E7.2.2.3; Na^+^-exporting ATPase |
| evm. model. Chromosome2.1870 | K21989 | TMEM63; calcium permeable stress-gated cation channel |
| evm. model. Chromosome7.601 | K22048 | MSL4S; mechanosensitive ion channel protein |
| evm. model. Chromosome5.1363 | K08994 | yneE, BEST; ion channel-forming bestrophin family protein |
| evm. model. Chromosome6.401 | K21864 | CCH1; voltage-dependent calcium channel |
| **Environmental resistance related genes** |  |  |
| evm. model. Chromosome1.2074 | K08158 | MDR1, FLR1, CAF5; MFS transporter, DHA1 family, multidrug resistance protein |
| evm. model. Chromosome3.439 | K03327 | TC. MATE, SLC47A, norM, mdtK, dinF; multidrug resistance protein, MATE family |
| evm. model. Chromosome1.1216 | K11811 | arsH; arsenical resistance protein ArsH |
| evm. model. Chromosome2.12 | K08158 | MDR1, FLR1, CAF5; MFS transporter, DHA1 family, multidrug resistance protein |
| evm. model. Chromosome1.1246 | K08158 | MDR1, FLR1, CAF5; MFS transporter, DHA1 family, multidrug resistance protein |
| evm. model. Chromosome5.1325 | K21249 | UVRAG; UV radiation resistance-associated gene protein |
| evm. model. Chromosome1.655 | K01483 | allA; ureidoglycolate lyase |
| evm. model. Chromosome2.1192 | K01091 | gph; phosphoglycolate phosphatase |
| evm. model. Chromosome5.72 | K13507 | glycerol-3-phosphate O-acyltransferase |
| evm. model. Chromosome3.470 | K06116 | glycerol-1-phosphatase |
| **Heat resistance related genes** |  |  |
| evm. model. Chromosome8.434 | K03283 | HSPA1s; heat shock 70kDa protein |

**Table S7** Results of secondary metabolite gene cluster alignment in the genome sequence of *S. rosea* A22

| Cluster Number | cluster type | similar gene clusters | similarity |
| --- | --- | --- | --- |
| Cluster1 | ectoine | ectoine | 100% |
| Cluster2 | bacteriocin | NF |  |
| Cluster3 | NRPS | marformycinA/marformycinB/ marformycinC/marformycinD/ marformycin E/marformycin F | 16% |
| Cluster4 | NRPS-like | NF |  |
| Cluster5 | T2PKS, hglE-KS | granaticin | 40% |
| Cluster6 | terpene | geosmin | 100% |
| Cluster7 | NRPS-like | paromomycin | 5% |
| Cluster8 | PKS-like, lanthipeptide | calicheamicin | 2% |
| Cluster9 | terpene | neoantimycin | 20% |
| Cluster10 | lanthipeptide | gentamicin | 4% |
| Cluster11 | T1PKS | versipelostatin | 20% |
| Cluster12 | NRPS, indole | methylpendolmycin/ pendolmycin | 42% |
| Cluster13 | other | mitomycin | 43% |
| Cluster14 | terpene | hopene | 46% |
| Cluster15 | other | NF |  |
| Cluster16 | NRPS-like | NF |  |
| Cluster17 | terpene | SF2575 | 6% |

**Table S8** Compatible solutes encoding genes in the genome of *S. rosea* A22

| Gene id | Gene | Protein |
| --- | --- | --- |
| A22_GM000370 | ectA | L-2,4-diaminobutyric acid acetyltransferase |
| A22_GM000371 | *ectB* | diaminobutyrate-2-oxoglutarate transaminase |
| A22_GM000372 | *ectC* | L-ectoine synthase |
| A22_GM000373 | *ectD* | ectoine hydroxylase |
| A22_GM000048 | *opuBD* | ABC transporter permease |
| A22_GM000049 | *opuA* | ATP-binding cassette domain-containing protein |
| A22_GM000050 | *opuBD* | ABC transporter permease |
| A22_GM000051 | *opuC* | glycine betaine ABC transporter substrate-binding protein |
| A22_GM002349 | *betB, gbsA* | gamma-aminobutyraldehyde dehydrogenase |
| A22_GM002969 | *betB, gbsA* | gamma-aminobutyraldehyde dehydrogenase |
| A22_GM004662 | *betB, gbsA* | gamma-aminobutyraldehyde dehydrogenase |
| A22_GM004912 | *betB, gbsA* | gamma-aminobutyraldehyde dehydrogenase |
| A22_GM001034 | *GDH2* | glutamate dehydrogenase |
| WP.029621430.1 | *gdhA* | NADP-specific glutamate dehydrogenase |
| A22_GM003957 | *gdhA* | glutamate dehydrogenase |
| A22_GM001916 | *TC.SSS* | sodium: solute symporter family protein |
| A22_GM003199 | *TC.SSS* | sodium: solute symporter family protein |
| A22_GM004642 | *TC.SSS* | sodium: solute symporter family |
| A22_GM005115 | *TC.SSS* | sodium: solute symporter family |
| A22_GM005207 | *TC.SSS* | sodium: solute symporter family |
| A22_GM004095 | *TC.SSS* | sodium: solute symporter family |
| A22_GM002179 | *cyc2* | germacradienol/geosmin synthase |

**Table S9** Differences of volatile flavor compounds in *huangjiu* brewed by wheat *Qu* inoculated with *Saccharopolyspora rosea* A22

| **Compounds** | **A22** | **Control** |
| --- | --- | --- |
| Ethyl acetate | 38.99±1.37^b^ | 46.87±1.46^a^ |
| Ethyl propionate | 0.16±0.02^a^ | 0.14±0.03^a^ |
| Ethyl propionate | 0.04±0.01^a^ | 0.07±0.01^a^ |
| Ethyl butyrate | 0.28±0.02^a^ | 0.24±0.03^a^ |
| Ethyl lactate | 22.71±1.66^b^ | 39.16±1.45^a^ |
| 2-Hydroxy-4-methyl-pentanoic acid ethyl ester | 0.04±0.01^a^ | 0.04±0.01^a^ |
| Ethyl isovalerate | 0.13±0.01^a^ | 0.18±0.02^a^ |
| Ethyl benzoate | 0.005±0.001^a^ | 0.004±0.001^a^ |
| Phenylethyl acetate | 0.013±0.002^a^ | 0.011±0.001^a^ |
| Ethyl phenylpropionate | 0.015±0.003^a^ | 0.013±0.001^a^ |
| **Esters** | **62.34±1.85**^b^ | **86.70±2.32**^a^ |
| N-butanol | 7.92±1.05^a^ | 8.36±0.59^a^ |
| N-hexanol | 0.68±0.18^a^ | 0.93±0.01^a^ |
| 1-octene-3-ol | 0.014±0.002^a^ | 0.014±0.002^a^ |
| **Alcohols** | **8.62±1.23**^a^ | **9.31±0.58**^a^ |
| Butyric acid | 9.81±0.34^a^ | 9.84±0.18^a^ |
| Isovaleric acid | 3.44±0.33^a^ | 3.15±0.15^a^ |
| 4-methylvaleric acid | 1.64±0.06^a^ | 1.69±0.01^a^ |
| Caproic acid | 2.74±0.02^a^ | 2.65±0.06^a^ |
| octoic acid | 0.888±0.002^a^ | 0.891±0.001^a^ |
| **Acids** | **18.52±0.75**^a^ | **18.21±0.21**^a^ |
| Isovaleraldehyde | 1.47±0.10^a^ | 1.66±0.14^a^ |
| Furfural | 1.90±0.20^a^ | 2.10±0.03^a^ |
| Quinaldehydes | 0.011±0.004^a^ | 0.008±0.001^a^ |
| 5-methyl furfural | 0.011±0.001^a^ | 0.014±0.002^a^ |
| Phenylacetaldehyde | 0.024±0.001^a^ | 0.023±0.001^a^ |
| **Aldehydes** | **3.42±0.31**^a^ | **3.80±0.14**^a^ |
| 4-vinyl guaiacol | 49.21±4.24^b^ | 70.54±2.29^a^ |
| guaiacol | 0.033±0.003^a^ | 0.024±0.002^a^ |
| phenol | 0.013±0.003^a^ | 0.006±0.001^b^ |
| 4-Ethylguaiacol | 0.025±0.001^a^ | 0.024±0.001^a^ |
| 4-Ethylphenol | 0.015±0.002^a^ | 0.016±0.001^a^ |
| **Phenols** | **49.30±4.25**^b^ | **70.61±2.29**^a^ |
| γ-Nonalactone | 0.033±0.001^a^ | 0.036±0.003^a^ |
